# Radiographic criteria in developmental dysplasia of the hip in late infancy, inter and intrareader agreement

**DOI:** 10.1371/journal.pone.0329230

**Published:** 2025-08-14

**Authors:** Desiree Alam, Souheil Hallit, Joseph Mandour, Boutros El Tannoury, Anthony Hassoun, Patrick Sami Bou Haidar, Majd El Hajj Moussa, Jean-Claude Lahoud, Charbel Tawk, Fadi Hoyek

**Affiliations:** 1 School of Medicine and Medical Sciences, Holy Spirit University of Kaslik, Jounieh, Lebanon; 2 Department of Psychology, College of Humanities, Effat University, Jeddah, Saudi Arabia; 3 Applied Science Research Center, Applied Science Private University, Amman, Jordan; 4 Department of Orthopedic Surgery, Notre Dame de Secours University Hospital Center, Byblos, Lebanon; King Abdulaziz University, SAUDI ARABIA

## Abstract

**Introduction:**

Developmental Dysplasia of the hip (DDH) is a common pediatric disorder screened for by antero-posterior (AP) pelvic radiographs in infants aged between 4–9 months. We chose from the radiographic indicators commonly used in the diagnosis the acetabular index, the Shenton line and the ossification and symmetry of the femoral head to assess for their reliability and variability among readers. In addition, this study aimed to obtain the mean age of appearance of the ossification center of the femoral head in the Lebanese population.

**Methods:**

149 pelvic AP radiographs of children between 4 and 9 months of age were collected. The criteria were assessed by three experienced readers: one orthopedic surgery fellow resident, one first-year and one second-year orthopedic surgery residents twice separated by a three-month interval.

**Results:**

The bivariate analyses found a difference in the right Acetabular angle and left Acetabular angle significantly in the first- and second-year resident with a p < 0.01. No significant difference was found when comparing the readings of each reader independently for the other variables or with the fellow. We found a significant difference p = 0.047 when comparing the readings of the first-year resident and the fellow of the right AI. Whereas the left AI readings revealed significant differences between the fellow and the second-year resident (p = 0.008) and between the first and second-year residents (p < 0.001). Inter and intrareader consistency was high for the Shenton line rupture and the appearance of the ossification center on the femoral head but none of the parameters proved sufficient to significantly be associated with an acetabular angle> 30°. The average age of ossification center appearance in the Lebanese population was determined to be 5.57 months, aligning with global averages.

**Conclusion:**

These findings call for a diagnostic approach that integrates multiple parameters and focuses on the importance of standardized training to enhance the consistency of radiographic assessments. Further investigations should aim to establish more precise protocols and evaluate the diagnostic strength of individual parameters.

## Introduction

Developmental Dysplasia of the Hip (DDH) is a common disorder in the pediatric population, with a prevalence of 3–4 per 1000 live births [1] and is known to be the most common musculoskeletal developmental disorder of the newborns [2].Its prevalence varies from a population to another where in Oman the reported incidence has been 1.05 per 1000 births [3] as compared to Greece with a prevalence of 10.83 per 1000 births [4]. It encompasses a broad spectrum of hip instability, ranging from acetabular or femoral dysplasia in a stable reduced hip, hip subluxation to total hip dislocation. It may appear at the time of birth or at a later stage, and was previously called “Congenital Dysplasia of the Hip” but the term was replaced by “Developmental” because the disease spectrum ranges from hip dysplasia to total dislocation [5]. Screening programs at the neonatal age have been set for over 30 years now but their precision remains debatable.

The diagnosis of DDH relies on the physical examination of the newborn, complemented by radiographic evidence to confirm and assess the severity of dysplasia, guiding therapeutic decisions [6]. The American Academy of Pediatrics and the Pediatric Orthopedic Society of North America recommend the use of the Barlow and Ortolani maneuvers for the screening of infants up to the age of 3 months [7], while other newer research relies of the limitation in hip abduction on clinical exam at the age of 8 weeks [7]. Ultrasound is the preferred modality before the appearance of the ossification center, typically for infants younger than 4 months, as the cartilaginous femoral head is not visible on radiographs during this period [6,8]. As for the assessment in infants older than 4 months, anteroposterior pelvic radiographs are used for systematic screening [6,8].

Although DDH is a prevalent disorder, with established radiographic criteria, the reliability of these methods is variable between studies and populations [1,6,9]. This disorder presents a slight increase in its prevalence for the past 30 years, some studies say [10],despite better screening and that can be possibly due to the changes in diagnostic criteria.

Inter and intra-reader agreement is therefore crucial especially when considering the widespread use of these criteria in the clinical setting and for research purposes. To standardize diagnostic purposes, we should be able to evaluate agreement in order to identify the variability in interpretation. This research emphasizes the assessment of inter- and intrareader consistency for three critical parameters: the acetabular angle, Shenton line, and the presence and symmetry of the ossification center. These criteria were selected due to their widespread use, quantifiability, and significance in evaluating the severity of DDH and informing treatment strategies [11]. Evaluating their reliability within the Lebanese pediatric population, that may have unique epidemiological patterns, aims to address diagnostic uncertainties and improve early identification efforts.

## Methods

### Human ethics and consent to participate

The Notre Dame des Secours University Hospital ethics committee approved the study protocol. The ethical committee waived the need to get the informed consent of the parents since it is a retrospective study. All methods were performed in accordance with the relevant guidelines and regulations (in accordance with the Declaration of Helsinki).

### Patient selection and data acquisition

149 anteroposterior (AP) supine pelvic radiographs were collected in a chronologic order including all the AP pelvic radiographs of patients between 4 and 9 months of age done systematically for screening in Lebanon, from the Notre Dame des Secours university hospital radiology department. The time range we included was from January 2021 till August 2023.Patients were excluded if they had prior hip surgeries or conservative interventions or if they did not fall in the age group margin and only the screening radiographs done systematically were included. Data collection was done by a medical student that does not know the patients’ clinical profile and were given to the readers anonymously and without the reports of the radiologists.

### Radiographic criteria

**Fig 1:**
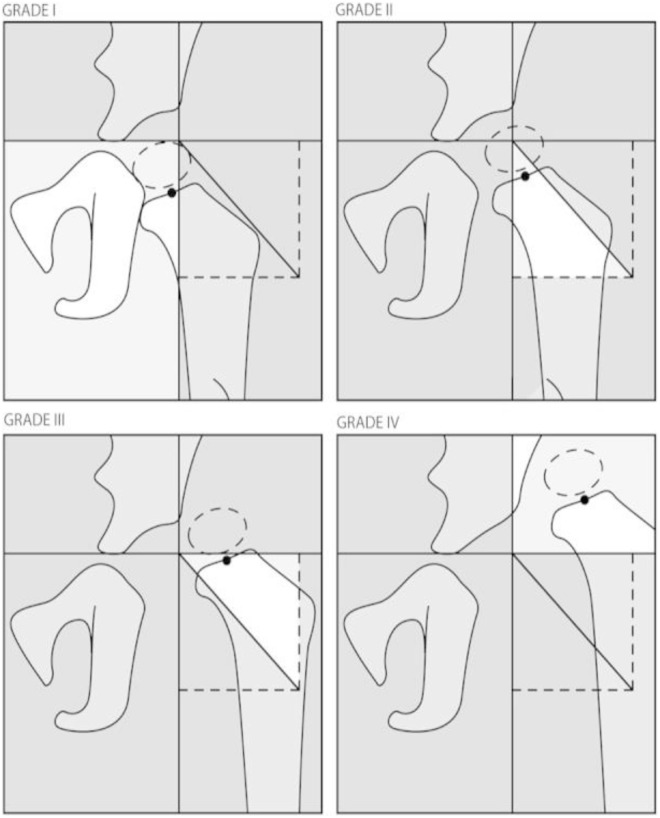
A. International Hip Dysplasia Institute Classification reference lines.

**Fig 2:**
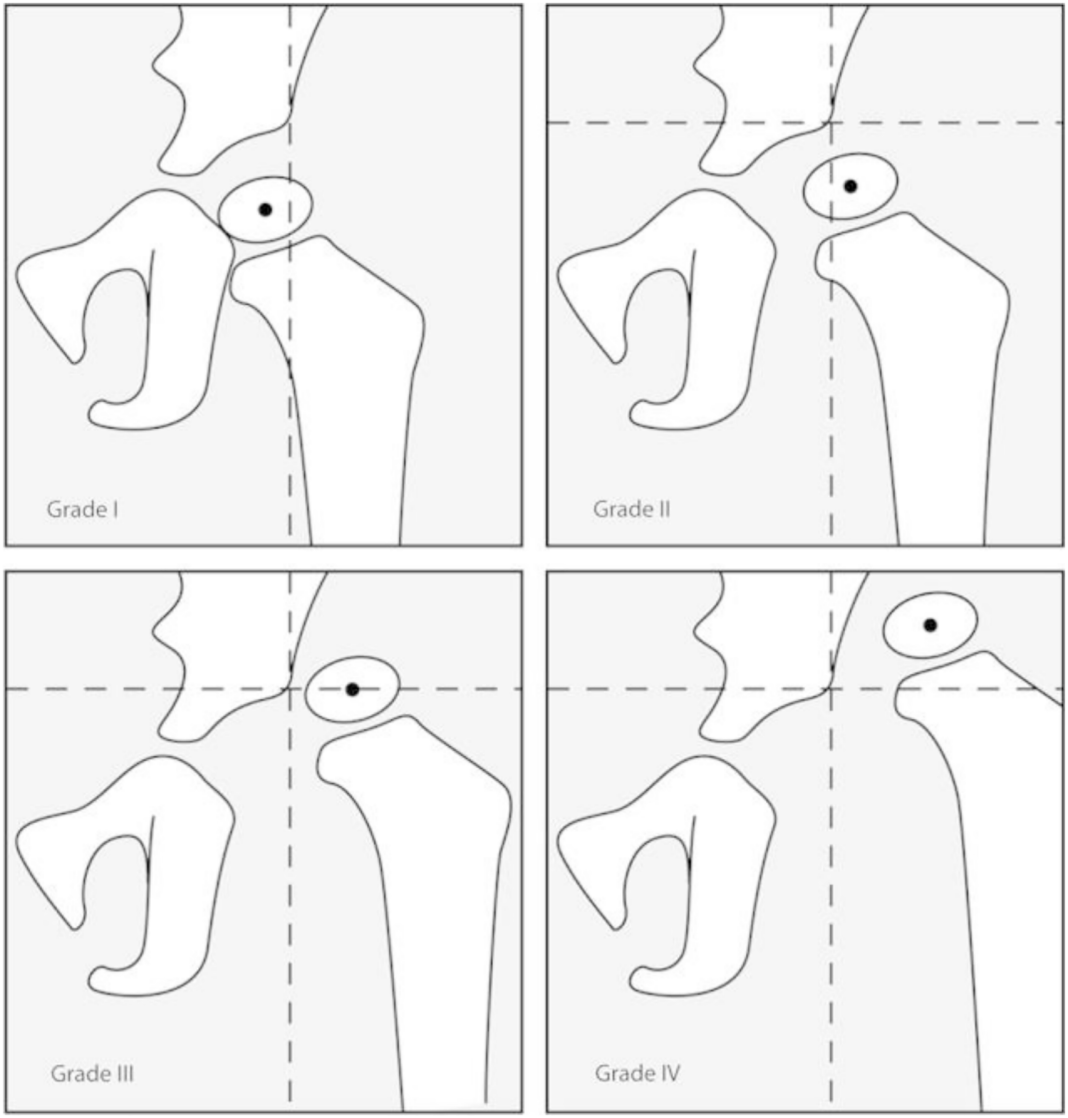
Tonnis Grade classification B, assigned grades by location of H point relative to reference point.

The lines used in the assessment and grading of this disorder in two of the most used classifications are illustrated in Figs 1, 2 [7,12] and 3 [2]. The figures are obtained from articles having the license BY CC 4.0 that allows the reuse of their published content.

**Fig 3:**
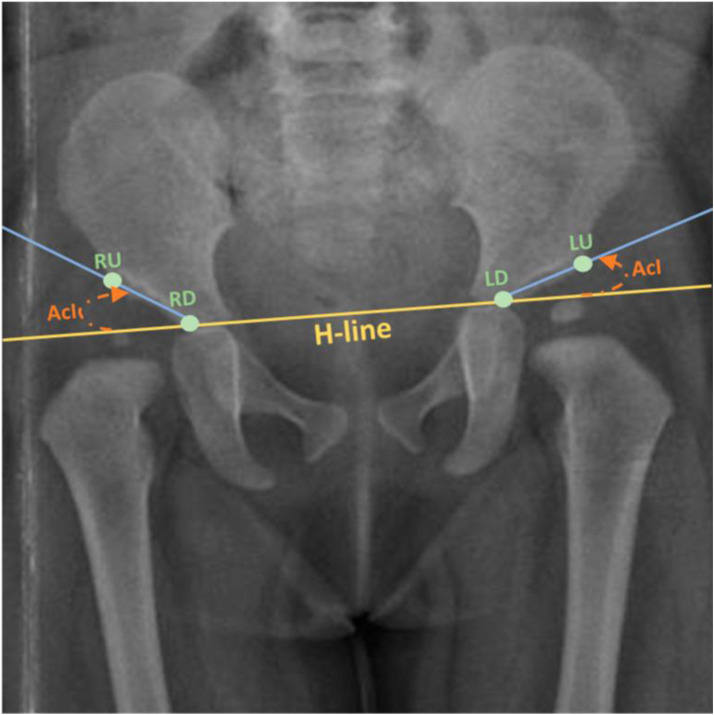
Calculating the AcI, with H-line displayed. Keypoints: LU: Left upper; LD: Left down; RU: Right upper; RD: Right down. [2].

One of the most important criteria in the assessment of DDH is the Acetabular Index, also known as Tonnis angle or Acetabular angle of Hilgenreiner, is a key radiographic criterion for DDH assessment. It is calculated in pelvic AP radiographs by measuring the angle between the Hilgenreiner line (H-line) (Fig 2), drawn at the top of the triradiate cartilages on both sides [9], and extending laterally to the supero-lateral end of the acetabular roof (Fig 3) [2]. It is used to evaluate the acetabular anatomy, knowing that in situations where the femoral head is not adequately aligned the acetabulum tends to flatten causing an increase in the acetabular angle [13]. This angle has been wide used as a reliable criterion in the diagnosis of DDH, the assessment of its severity and to determine the efficacy of the management [14]. The normal angle should not exceed 30° in a neonate and decreases to ≤22° by the age of 1 year [9]. An increase in the acetabular inclination range “AcI” is strongly indicative of a DDH [2]. Furthermore, the Shenton line, described in 1911 by Edward Warren Hine Shenton, represents the projected arc between the inferior border of the femoral neck to the superior border of the obturator foramen [15]. Disruption in the Shenton line suggests the possibility of subluxation or dislocation of the femoral head from the acetabulum, strongly indicative of a dysplastic acetabulum [15].

The ossification center plays a significant role in the diagnosis of DDH, particularly after the neonatal period. Delayed or asymmetric development of the ossification center is a hallmark of DDH, reflecting impaired growth in the affected hip [16]. By correlating the appearance of the ossification center with age, this study aims to identify population-specific patterns that may improve diagnostic accuracy and timing of interventions.

### Radiographs analysis

The radiographs were done using a single Shimadzu X-ray machine and followed the same acquisition protocol. The infants were put in a supine position with a flat pelvis and the legs gently internally rotated. The exposure is automatically adjusted by the machine based on the age and the age and the infant’s size with average values of 63–70 kVp, 2–5 mAs and 100 cm SID. All radiographs were collimated to skin margins above iliac crests and below the proximal femurs.

One orthopedic fellow, one second-year orthopedic resident and a first-year orthopedic resident were the readers in this study. They were selected by the head of department of orthopedic surgery after their approval to participate in our study based on his judgment of their knowledge, training and interest in pediatric orthopedic surgery and hip diseases. They were given blindly the radiographs and a questionnaire in which they had to fill for each radiograph the following: (1) The measurement of the Acetabular index on the right and the left hip joint, (2) The presence or the absence of a ruptured Shenton Line bilaterally, and (3) The presence or absence of the ossification center on the right and on the left and its symmetry. These readings were reperformed after 3 months by the same readers and over the same radiographs.

### Patients’ classification

Based on the radiologists’ reports patients with DDH were identified as those having an acetabular index > 30° considered as the upper limit of the normal based on the literature review [5] after checking with an experimented pediatric orthopedic surgeon.

### Statistical analysis

The SPSS software v.27 was used for the statistical analysis. The right and left acetabular angles were considered normally distributed for the skewness and kurtosis values varied between −1 and +1. We used the Chi-2 test to compare two categorical variables and the ANOVA test to compare three means; the Bonferroni post-hoc test was used to compare the readers categories two by two. The paired t test was used to compare the first and second readings respectively. Finally, the age variable was not considered normally distributed; consequently, the Mann-Whitney test was used to compare the mean age between the presence of ossification center or not. To assess intra- and inter-rater reliability, the Intraclass Correlation Coefficient (ICC) was calculated using a two-way random effects model with consistency type. Values of ICC range from 0 to 1, with higher values indicating better reliability. An ICC value below 0.5 indicates poor reliability, 0.5–0.75 moderate reliability, 0.75–0.9 good reliability and > 0.9 excellent reliability. All ICCs were reported with 95% Confidence Intervals. P < 0.05 was deemed statistically significant.

## Results

A total of 460 questionnaires were filled, with a mean age of 5.35 months. All participant characteristics can be found in Table 1.

**Table 1:** Sociodemographic characteristics of participants (N = 460).

	N (%)
**Reader**	
** **Fellow	149 (32.4%)
** **Second year resident	149 (32.4%)
** **First year resident	162 (35.2%)
	**Mean ± SD**
**Age (months)**	5.35 ± 1.18

Bold numbers indicate significant p value. ICC = Intraclass Correlation Coefficient.

### Bivariate analyses

The results of the paired t test conducted on the total sample showed an overall difference in the reading of the right acetabular angle in the total sample (22.83 vs 22.61; p < 0.001). No difference was found in the fellow’s reading of the 3 variables (Table 3), whereas a difference in the right Acetabular angle and left Acetabular angle was significantly found in the first- and second-year resident (Tables 4 and 5 respectively).

**Table 2:** Comparison of the difference in the right and left Acetabular angle between the first and second reading between the readers.

Variable	Difference right Acetabular angle				Difference left Acetabular angle			
	Mean ± SD	P	Effect size	ICC [95% CI] (Right)	Mean ± SD	P	Effect size	ICC [95% CI] (Left)
**Reader**		**<0.001**	0.016			**<0.001**	0.044	
** **Fellow	0.00 ± 0.00			1.00 [1.00; 1.00]	0.01 ± 0.25			0.999 [0.999; 0.999]
** **Second year resident	0.33 ± 0.75			0.992 [0.989; 0.994]	0.46 ± 1.44			0.978 [0.969; 0.984]
** **First year resident	0.34 ± 1.93			0.947 [0.928; 0.961]	−0.19 ± 1.66		0.175	0.965 [0.952; 0.974]

Bold numbers indicate significant p value. ICC = Intraclass Correlation Coefficient.

**Table 3:** Differences of the dichotomized right and left Acetabular angle among readers.

Variable	First reading Mean ± SD	Second reading Mean ± SD	*p*	Effect size
**Model 1: Fellow**				
**Right Acetabular angle Yes/No**	0.03 ± 0.16	0.03 ± 0.16	–	–
**Left Acetabular angle Yes/No**	0.10 ± 0.30	0.10 ± 0.30	–	–
**Model 2: Second-year resident**				
**Right Acetabular angle Yes/No**	0.04 ± 0.20	0.06 ± 0.24	0.083	0.143
**Left Acetabular angle Yes/No**	0.08 ± 0.27	0.09 ± 0.29	0.319	0.082
**Model 3: First-year resident**				
**Right Acetabular angle Yes/No**	0.07 ± 0.25	0.04 ± 0.20	0.103	0.129
**Left Acetabular angle Yes/No**	0.06 ± 0.24	0.06 ± 0.24	1	0.001

**Table 4:** Comparison of the difference integrity in the right and left Shenton’s Line between the first and second reading between the readers.

Variable	Integrity difference of the right Shenton Line				Integrity difference of the left Shenton Line			
	Mean ± SD	P	Effect size	ICC [95% CI] (Right)	Mean ± SD	P	Effect size	ICC [95% CI] (Left)
**Reader**								
** **Fellow	0.00 ± 0.00			1.00 [1.00; 1.00]	0.00 ± 0.00			1.00 [1.00; 1.00]
** **Second year resident	0.01 ± 0.08			1.00 [1.00; 1.00]	0.01 ± 0.08			1.00 [1.00; 1.00]
** **First year resident	0.01 ± 0.08			1.00 [1.00; 1.00]	0.01 ± 0.08			1.00 [1.00; 1.00]

**Table 5:** Comparison of the difference in localizing the right and left ossification centers of the femoral head and their symmetry between the first and second reading between the readers.

Variable	First reading Mean ± SD	Second reading Mean ± SD	*p*	Effect size	ICC [95% CI]
Model 1: using the total sample					
**Right ossification center**	0.62 ± 0.49	0.62 ± 0.49	0.318	0.047	0.998 [0.997; 0.998]
**Left ossification center**	0.62 ± 0.49	0.62 ± 0.49	0.318	0.047	0.998 [0.997; 0.998]
**Symmetry**	0.70 ± 0.46	0.70 ± 0.46	1	0.001	0.794 [0.753; 0.828]
Model 2: In the fellow					
**Right ossification center**	0.65 ± 0.48	0.65 ± 0.48	–	–	1.00 [1.00; 1.00]
**Left ossification center**	0.66 ± 0.47	0.66 ± 0.47	–	–	1.00 [1.00; 1.00]
**Symmetry in the ossification centers**	0.62 ± 0.49	0.62 ± 0.49	–	–	1.00 [1.00; 1.00]
Model 3: In the second-year resident					
**Right ossification center**	0.65 ± 0.48	0.66 ± 0.48	0.319	0.082	0.993 [0.990; 0.995]
**Left ossification center**	0.65 ± 0.48	0.66 ± 0.48	0.319	0.082	0.993 [0.990; 0.995]
**Symmetry in the ossification centers**	0.61 ± 0.49	0.62 ± 0.49	1	0.001	0.128 [−0.205; 0.369]
Model 4 In the first-year resident					
**Right ossification center**	0.57 ± 0.50	0.57 ± 0.50	–	–	1.00 [1.00; 1.00]
**Left ossification center**	0.55 ± 0.50	0.55 ± 0.50	–	–	1.00 [1.00; 1.00]
**Symmetry in the ossification centers**	0.86 ± 0.35	0.86 ± 0.35	–	–	1.00 [1.00; 1.00]

Bold numbers indicate significant p value. ICC = Intraclass Correlation Coefficient.

#### 1- Acetabular angle measurements.

When comparing the difference in the right and left Acetabular angles between the first and second reading between the readers, the results showed a significant difference between the readers for both Acetabular angles (p < 0.001 for both) (Table 2). The Bonferroni post-hoc test results showed a significant difference of the reading of right Acetabular angle between the fellow and the first-year resident (p = 0.047) but not between the fellow and the second-year resident (p = 0.064) or between the second-year and first-year resident (p = 1). Moreover, a significant difference was found between fellow and second-year resident for left Acetabular angle measurements (p = 0.008), between second-year and first-year resident (p < 0.001) but not between the fellow and the first-year resident (p = 0.484).

When comparing the dichotomized right and left acetabular angles (yes/no) in the total sample to address any possible variability in recognizing the acetabular angle >30°, no significant difference was found between the first and second reading in terms of right acetabular angle measurement (0.05 ± 0.21 vs 0.04 ± 0.20; p = 0.739; Effect size = 0.016) and left acetabular angle measurement (0.08 ± 0.27 vs 0.08 ± 0.28; p = 0.480; Effect size = 0.033). No significant difference was found in each reader separately (Table 3).

#### 2- Shenton line integrity.

Shenton line rupture was extremely rare in both right and left hips, with almost all readings indicating integrity across all readers and both readings. The results are presented in Table 4.

#### 3- Ossification center presence and symmetry.

No significant difference was found when comparing the two readings of each reader when localizing the ossification center of the femoral head and its symmetry on both hips (Table 5).

When comparing the acetabular angle >30° with the visibility of the ossification center, no significant differences were observed between the right acetabular angle and the presence of the right ossification center in both the first and second readings (Tables 6a and 6b). However, a higher percentage of patients with a visible left ossification center was noted among those with a left acetabular angle >30° in both the first and second readings (Tables 6c and 6d).

**Table 6:** Cross-tabulation. between Acetabular angle and ossification center.

6A. Cross-tabulation between right Acetabular angle and right ossification center in the first reading.				
Variable	Right Acetabular angle 1		*p*	Effect size
**Right ossification center 1**	**No**	**Yes**	0.650	0.023
** No**	165 (94.8%)	9 (5.2%)		
** Yes**	274 (95.8%)	12 (4.2%)		
**6B. Cross-tabulation between right AC and right ossification center in the second reading.**				
**Variable**	**Right Acetabular angle 2**		***p***	**Effect size**
**Right ossification center 2**	**No**	**Yes**	0.495	0.033
** No**	167 (96.5%)	6 (3.5%)		
** Yes**	273 (95.1%)	14 (4.9%)		
**6C. Cross-tabulation between left AC and left ossification center in the first reading.**				
**Variable**	**Left Acetabular angle 1**		***p***	**Effect size**
**Left ossification center 1**	**No**	**Yes**	**0.013**	0.117
** No**	168 (96%)	7 (4%)		
** Yes**	255 (89.5%)	30 (10.5%)		
**6D. Cross-tabulation between left Acetabular angle and left ossification center in the second reading.**				
**Variable**	**Left Acetabular angle 2**		***p***	**Effect size**
**Left ossification center 2**	**No**	**Yes**	**0.024**	0.109
** No**	166 (95.4%)	8 (4.6%)		
** Yes**	255 (89.2%)	39 (8.5%)		

### Comparison of age in terms of the ossification center

A significantly higher mean age was found in patients with right and left ossification center found in the first and second reading respectively compared to not (p < 0.001 for all comparisons) (Table 7).

**Table 7:** Comparison of age in terms of the ossification center.

Variable	Age		
	Mean ± SD	P	Effect size
**Right ossification center – First reading**		**<0.001**	0.516
No	4.98 ± 0.91		
Yes	5.57 ± 1.26		
**Right ossification center- Second reading**		**<0.001**	0.549
No	4.96 ± 0.86		
Yes	5.58 ± 1.28		
**Left ossification center- First reading**		**<0.001**	0.484
No	5.00 ± 0.90		
Yes	5.56 ± 1.27		
**Left ossification center- Second reading**		**<0.001**	0.516
No	4.98 ± 0.85		
Yes	5.57 ± 1.29		

## Discussion

DDH is a common pediatric disorder screened for in children aged between 4 and 9 months by plain AP radiographs of the pelvis, therefore the reliability of the measurement tools used is of major importance to ensure the standardization of diagnosis. We chose from these criteria the acetabular angle measurement, the presence and symmetry of the ossification centers of the femoral heads and the rupture or continuity of the Shenton Line. In previous studies, like the one conducted by Yan et al., the ossification center appeared radiologically by the age of 4–6 months in normal hips [16], consistent with the result we obtained in our population where the mean age of its appearance is 5.57 months. Our study found no statistically significant association between femoral head ossification symmetry and the acetabular index superior to 30° contrasting with the same study by Yan et al. [16] that revealed a delayed ossification in dysplastic hips. This discrepancy has originated from population variabilities (e.g., age, severity of dysplasia) or from sample size. However, consistently with Upasani et al. [4,17] our high intrareader agreement highlights that ossification delay can be an obstacle in front of accurate radiologic landmark positioning what was considered as a limitation for the Tonnis classification, this finding suggests that the ossification symmetry is a consistent marker but its utility remains questionable before the age of 6 months where delayed ossification is common even in normal hips.

On another hand, our findings suggested that the Shenton’s line was intact in the majority of hips, even in cases where the acetabular angle exceeded 30°,consistent with Reiman et al. [17,18] who reported that that the Shenton’s line disruption rises the likelihood of hip dysplasia from its baseline of 17%−50% to a much higher 77%−91% which means its positivity strongly suggests dysplasia, whereas the negativity or continuity of the same line decreases the likelihood of dysplasia but to a lesser extent from 20% to 15% so an intact Shenton’s line reduces the chance of dysplasia but does not rule it out completely. This supports its role as a “red flag” but not a standalone diagnostic tool. Furthermore, there was no significant difference between the first and second readings among the three readers, suggesting that while the Shenton’s line may demonstrate accuracy, it lacks reliability as a standalone diagnostic tool for DDH. [18].

Moreover, the acetabular index, one of the most important radiologic criteria for the diagnosis of DDH, has also been criticized for having high rates of inter and intra-observer variabilities [17]. Some studies found good to excellent agreement results [17,19]. While other studies focused on the difficulty in identifying the radiographic anatomic landmarks being a limitation to the accuracy of the measurement of this angle [17], what gave rise to different alternative measurements trying to minimize these differences [14]. Another study conducted by Upasani et al. has found a questionable reproducibility in the measurement of the acetabular index, especially when important pelvic landmarks have not ossified yet, especially the femoral head and the lateral aspect of the acetabulum [17]. In this study, a significant difference in the interreader reproducibility in the junior residents contrasts with the findings of Elifranji et al. [20] that found excellent interclass correlation coefficient (ICC > 0.95) among trainees. This underscores the importance of standardized training; their readers underwent landmark localization training workshops whereas our residents relied on self-guided practice, noting that in the same study, they concluded that the workshops reduced variabilities by 30% [20]. Whereas the interreader reliability of the fellow resident was very high and this can attributable to the difference in the experience interpreting radiograph gained throughout the residency program. However, when comparing the intrareader agreement concerning the measurement of the acetabular index, no significant difference was found making this reading accurate regardless of the slight differences that can be present. This finding is probably due to consistency in the localization of landmark points that minimizes variability regardless of the level of expertise, or because of familiarization with pelvic radiographs after two readings that reduced the risk of inconsistencies.

### Limitations

This study has several limitations that could have affected the results. Only three readers selected by the head of department based on his assessment of their ability to read and interpret hip radiographs, have performed the measurements and their variable experiences could have been less impactful if more readers participated. This factor could have predisposed us to selection bias. Although these readers have important knowledge in reading pelvic radiographs and the inter-reader agreement on the measurements was mostly high, the participation of experienced radiologists or orthopedic surgeons might have decreased the standard error of the study results. The number of readings requested from each participant to be done twice and the amount of time it consumed might have been a burden to the physicians and have caused a decrease in the accuracy of their readings. In addition, the sample size was relatively small and taken from a single medical center to be able to generalize the results obtained to the Lebanese or international population. Additional parameters that were not included in this project could have also influenced the quality of the results, such as gender, reader experience and radiograph quality.

## Conclusion

This study highlights the complexity of reliably in the diagnosis of developmental dysplasia of the hip (DDH) using common radiographic markers in infants. Our findings demonstrate that the acetabular angle, Shenton’s line, and femoral head ossification center have notable limitations in consistency and diagnostic value.

Although mean acetabular angle values were within expected ranges, significant intra- and inter-observer variability was observed, particularly among less experienced readers. The fellow showed consistent measurements, while first- and second-year residents presented significant differences between repeated readings, especially for the acetabular angle (p < 0.001). These findings highlight the influence of reader experience on measurement reliability. The rupture of Shenton’s line was rare (<1% of cases), limiting its practical utility as a routine screening marker in this specific age group. The presence and symmetry of the femoral head ossification center correlated significantly with age (p < 0.001), but were not with the measurements of the acetabular angle. As a summary, no single marker or combination provided a definitive diagnosis of DDH, highlighting the necessity of a comprehensive approach that integrates clinical assessment with multiple imaging parameters. This study supports the integration of standardized training protocols and multi-criteria evaluation to improve the accuracy and consistency of DDH diagnosis in infants, ultimately improving early detection and management strategies. Our study results open to the door in front of future research projects aiming to find the best diagnostic protocol for DDH diagnosis probably including a larger sample size or a multicentral study hoping to obtain standardized guidelines. Now with the presence of the Artificial intelligence we can work as well on using the technology while interpreting radiographs aiming to minimize human error and obtain more consistent measurements.
